# A Computational Study of Spike Time Reliability in Two Types of Threshold Dynamics

**DOI:** 10.1186/2190-8567-3-11

**Published:** 2013-08-14

**Authors:** Yue-Xian Li, Rachel Kuske

**Affiliations:** 1 Department of Mathematics, University of British Columbia, Vancouver, BC, Canada, V6T 1Z2; 2 Department of Cell Biology and Anatomy, Louisiana State University Health Sciences Center, New Orleans, LA, 70112, USA

**Keywords:** Spike triggered average, Neuronal excitability, Stochastic reliability

## Abstract

Spike time reliability (STR) refers to the phenomenon in which repetitive applications of a frozen copy of one stochastic signal to a neuron trigger spikes with reliable timing while a constant signal fails to do so. Observed and explored in numerous experimental and theoretical studies, STR is a complex dynamic phenomenon depending on the nature of external inputs as well as intrinsic properties of a neuron. The neuron under consideration could be either quiescent or spontaneously spiking in the absence of the external stimulus. Focusing on the situation in which the unstimulated neuron is quiescent but close to a switching point to oscillations, we numerically analyze STR treating each spike occurrence as a time localized event in a model neuron. We study both the averaged properties as well as individual features of spike-evoking epochs (SEEs). The effects of interactions between spikes is minimized by selecting signals that generate spikes with relatively long interspike intervals (ISIs). Under these conditions, the frequency content of the input signal has little impact on STR. We study two distinct cases, Type I in which the *f*–*I* relation (*f* for frequency, *I* for applied current) is continuous and Type II where the *f*–*I* relation exhibits a jump. STR in the two types shows a number of similar features and differ in some others. SEEs that are capable of triggering spikes show great variety in amplitude and time profile. On average, reliable spike timing is associated with an accelerated increase in the “action” of the signal as a threshold for spike generation is approached. Here, “action” is defined as the average amount of current delivered during a fixed time interval. When individual SEEs are studied, however, their time profiles are found important for triggering more precisely timed spikes. The SEEs that have a more favorable time profile are capable of triggering spikes with higher precision even at lower action levels.

## 1 Introduction

A constant current applied to a neuron at different times usually triggers trains of spikes that do not show reliable timing, due probably to the effects of intrinsic noise and/or differences in the initial state of the neuron when the signal is turned on each time. A stochastically fluctuating signal, however, is capable of generating spikes with remarkably reliable spike timing [1]. This phenomenon has been called spike time reliability (STR) [2], and has been widely observed experimentally in a number of different neurons [1-6] and investigated in several theoretical studies [7-10]. Known as a general property of spiking model neurons [11], STR is closely related to the study of synchronization of uncoupled periodic or chaotic attractors driven by a common noise (see, e.g., [12]). 

Given the connection of STR both to the precise mapping of stimuli onto responses and to synchronization, a variety of settings have been explored to better understand the mechanisms and signal features that facilitate STR. For example, gamma range fluctuations have been shown to facilitate the generation of more precisely-timed spikes and induce higher variability in interspike intervals (ISIs) [3]. Effects of the frequency content and the relative amplitude of periodic fluctuations on STR have been investigated in [4,5]. [11] showed that aperiodic inputs, contrary to periodic ones, induced reproducible responses. Reliability in the timing of bursts of action potentials can also be achieved through a frozen random input [13]. Galan et al. showed in both experiments and simulations that STR exhibits a local maximum as the correlation time of the external input is increased. In an apparently different context of stochastic resonance, a mechanistically related phenomenon was also demonstrated in a summing network of excitable units [14]. 

Even with the large range of studies of STR available, there are certain aspects that have received less exploration. By considering the neural behavior without an external stimulus, we can identify two general situations for STR that have received significantly different levels of attention. In the first situation, which has been considered widely, the neuron is spontaneously spiking and exhibits self-sustained oscillatory activities in the absence of external stimulation. In this case, the frequency component of the external noise has been shown to be important for STR [4,5]. For uncoupled spontaneous oscillators, [12,15] showed that synchrony driven by a common noise is associated with the emergence of a negative leading Lyapunov exponent in analytical studies, taking advantage of the phase theory. In the second situation, for which there are considerably fewer results available, the neuron is quiescent in the absence of external noise. A number of different phenomena can occur in this situation. For example, the introduction of noise could induce coherence resonance [16], thus turning the neuron into a noise-induced coherent oscillator. Analytical results in [17] provide an in-depth understanding of noise-induced transitions from quiescence to oscillations, but these did not consider STR. A computational study using conductance based models compared STR and its precision both in the mean-driven firing range [18], where firing occurs without an external stimulus, and in the fluctuation-driven regime, where firing occurs only with an external stimulus that drives threshold crossing. There the influences of frequency and amplitude of oscillatory stimuli are emphasized, and conditions where impedance profiles may be important were identified. In a recent theoretical study, STR in the case of quiescent neurons is analyzed [19]. There stochastic amplitude and phase equations for two coupled canonical conditional oscillators were derived in a subthreshold parameter regime and under the influence of distinct intrinsic noises and a common external stochastic drive. The asymptotic approximations for the probability density of the phase difference revealed that, for dominant common extrinsic noise, the phase difference is strongly peaked at zero for comparable intrinsic noise levels or at a nonzero phase difference for different levels of intrinsic noise, indicating synchronized or phase-locked oscillations. In addition, the spikes generated by the common external noise could appear irregular and incoherent, yet spike timing is remarkably precise when a frozen copy of the external noisy stimulus is applied, as observed in [1]. In the setting where the neurons are quiescent without an external noisy stimulus, we have not yet seen thorough theoretical or mechanistic explanations for the range of elements that can promote STR. The present study is aimed at revealing some important aspects of such mechanisms using a simple neuronal model. 

For a more complete understanding of the STR in a quiescent neuron, in the context of noise-induced irregular and incoherent spiking, it is necessary to determine which features of the input signal are crucial for triggering spikes with precise timing. Spike initiation in a quiescent neuron is often associated with a threshold phenomenon, which happens when a critical transmembrane potential is exceeded. Therefore, spike-triggering can be regarded as a complex “pattern recognition” process [1], a “feature detection/dimensional reduction” process [20,21], or even a “time-localized resonance” process, as we view it here and in [22], providing support for this concept in this paper. Mathematical models of neurons are useful tools in exploring these aspects of STR. Unlike real neurons in which the dimensionality and the intrinsic noise are both unknown, the dimension of a model and the intrinsic noise are well defined in mathematical models. We carry out a computational analysis of the Morris–Lecar model [23], which has only two variables. Both the intrinsic noise and external signals enter through the voltage equation. This model allows an analysis of the threshold of spike-generation in the phase plane in order to investigate previously unexplored aspects of the external stimulus that can support STR. An understanding of both the phase-plane dynamics and bifurcation of the underlying system contributes to the identification of the key elements of the reliable spike-evoking epochs (SEEs) in the signal. The dynamical analysis is particularly important in the context of quiescent neurons, where features like intrinsic frequencies or well-defined regular oscillation are not necessarily contributing factors as they are when the neurons exhibit repetitive firing in the absence of an input signal. 

STR in the context of our study is closely related to three important factors of the external noisy input signal: (i) the ability of external fluctuations to eliminate the memory of accumulated variations in the neuron; (ii) the ability of a fluctuation to trigger a spike reliably in the presence of different copies of intrinsic noise; and (iii) the potentiation of a time localized temporal epoch in the input that “resonates” with the neuron to trigger a spike reliably at low amplitude. Focusing on these factors allows us to identify the key features of individual spike-evoking epochs (SEEs) that drive reliable spike timing, even though these SEEs show great variety in amplitude and time profile. On average, two specific features are shown to be crucial for reliable spike timing: (1) the accelerated increase in “action” level (defined as the amount of current delivered during a fixed time interval) as the spiking threshold is approached and (2) the time profile of a SEE. The SEEs that have a more favorable time profile trigger more precise spike timing at a reduced level of action. These results are in good agreement with experimental observations [1]. 

The effects of the three key factors mentioned above are best studied when the effects of other dynamic properties of a neuron are minimized, such as the interspike refractory period (IRP) and the intrinsic frequency of an oscillatory neuron. It has been shown that STR is reduced when the frequency of the stimulus-induced response is high [6], typically due to interference with the IRP or intrinsic frequency. To minimize the influence of IRP and intrinsic frequencies on model neurons that are quiescent in the absence of external inputs, we use specifically generated input signals that only trigger spike trains with relatively long ISIs. An important part of the input signals are spike-triggered averages (STAs), obtained from averaging many time-varying spike-generating stimuli over small time windows preceding the spikes [24]. Using STAs rather than signals with a specific frequency is necessary for our setting, since the dynamics of the quiescent neuron is not associated with a specific intrinsic frequency. In fact, we show that the reliability is insensitive to frequency content of the noisy signal, as long as the ISIs are sufficiently long. 

The phase-plane analysis presented here indicates how the combination of action and time profile leads to reliable spike timing in the setting of quiescent neurons. This analysis is complemented by studying both the averages and distributions of properties of the SEEs and the STAs obtained from subsets of the SEEs. Additional important elements are suggested by the characteristics of different subsets of the SEEs that are found to be reliable, such as a time profile that allows the system to “settle” near the steady state while providing sufficient current over a time interval. In combination, these characteristics contribute to the three important factors mentioned above, and increase the precision of the firing over repeated trials.

Furthermore, we see that the underlying bifurcation structure of the neuron, which characterizes its quiescence-to-spiking transition, can also influence the effectiveness of the action-time profile combination. We study two distinct sets of parameter values that give rise to two bifurcation types, and thus two different scenarios of spike transition. Type I is characterized by a gradual increase in the frequency from zero as the bifurcation parameter—here the (base) input current—changes beyond the transition point. Type II is characterized by a jump increase in the oscillation frequency as the bifurcation parameter reaches the quiescence-to-spiking transition point (see [25,26] for details about Type I and Type II neurons). The behavior in the spiking transition is considered in connection with the slow- (or pseudo-slow) manifold in Type I (or II), and related to the firing threshold for each type. The significance of a manifold, rather than a single point, in firing thresholds has been recognized in a range of contexts, starting with FitzHugh [27], and more recently in the areas of excitability, bursting, and mixed-mode oscillations [28,29]. The relationship of different neuron dynamics to phase plane structures that approximate the firing threshold has been developed as a diagnostic tool to understand variable neuron responses to fluctuating environmental parameters (see, e.g., [30] and references therein). Here, we compare the transitions and the underlying threshold dynamics in the phase plane, to reveal the differences and similarities in the roles that action and time profile play in the two types. For example, in Type I neurons the fluctuations near the slow manifold allow for reliable spiking transitions over a smaller range of action levels, with greater variation in the precision. Thus, we indicate the role that the bifurcation structure can play in identifying features that support STR. 

The remainder of the paper is organized as follows. In the model and methods section, we introduce the Morris–Lecar model that we used in the simulations. We discuss the measure we employed to calculate the reliability of spike timing, as well as the computational methods employed in the simulation and analysis. The main results are presented in the results section that is followed by the discussion section.

## 2 Model and Methods

### 2.1 The Model

We use the Morris–Lecar (ML) model [23] in the present study. The noise terms are included additively to the voltage equation.

(2.1) $$\begin{array}{rcl} c\frac{dv}{dt} & = & -g_{\mathrm{Ca}}m_{\infty }(v-v_{\mathrm{Ca}})-g_{K}w(v-v_{K})-g_{L}(v-v_{L})\\ & & +I_{\mathrm{bias}}+\delta_{1}\eta_{1}(t)+I_{\mathrm{ext}},\\ \frac{dw}{dt} & = & \lambda (v)\left(w_{\infty }(v)-w\right), \end{array}$$

where *v* is the membrane voltage potential; *w* represents the probability of opening of K^+^ channels (0≤w≤1). $m_{\infty }(v)=0.5(1+\tanh((v-v_{1})/v_{2}))$, $w_{\infty }(v)=0.5(1+\tanh((v-v_{3})/v_{4}))$, $\lambda (v)=\phi \cosh((v-v_{3})/(2v_{4}))$. Here, $I_{\mathrm{bias}}$ is a base or bias current that is constant in the neuron, typically viewed as the underlying fixed control or bifurcation parameter related to different states in the dynamics. The term $\delta_{1}\eta_{1}(t)$ is the intrinsic noise, where $\eta_{1}(t)$ is modeled as a standard Brownian motion, with a distinct realization of the intrinsic noise used on each trial. We refer to the coefficient $\delta_{1}$ to indicate the intensity of the intrinsic noise. The term $I_{\mathrm{ext}}$ is an additional extrinsic current, given by $I_{\mathrm{ext}}=I_{c}+\delta_{2}\eta_{2}$, where the constant $I_{c}$ is just a shift of $I_{\mathrm{bias}}$, and $\delta_{2}\eta_{2}(t)$ is a stochastic current. The coefficient $\delta_{2}$ is a constant and the contribution $\eta_{2}(t)$ is obtained by convolving a Gaussian white noise and an alpha function $\alpha (t)=(t/\tau^{2})\exp(-t/\tau )$ with a time constant *τ*. Therefore, the actual standard deviation (SD) of the external noise $\delta_{2}\eta_{2}(t)$ is $\delta_{2}\sigma_{2}$ where $\sigma_{2}$ is the SD of $\eta_{2}(t)$. In the rest of this paper, we use the SD to refer to the intensity of the extrinsic noise. Note that the extrinsic signal $I_{\mathrm{ext}}$ is a frozen copy of a noisy external input used across trials.

**Fig. 1:**
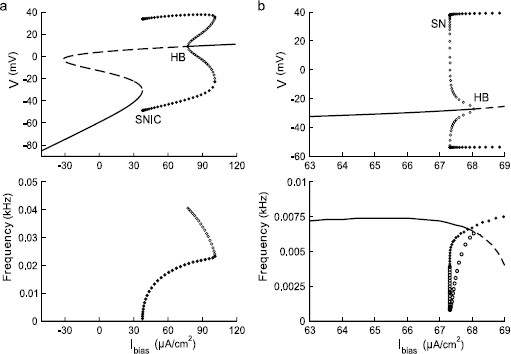
Bifurcation diagrams and the corresponding *f*–$I_{\mathrm{bias}}$ relationship near a Type I (**a**) and a Type II (**b**) transition in the Morris–Lecar (ML) model. $I_{\mathrm{bias}}$ is the control parameter. Stable and unstable equilibria are marked with *solid* and *dashed lines*, respectively. Stable and unstable periodic solutions are marked with *filled* and *open circles*, respectively. The intrinsic frequency of a steady state is determined by the imaginary part of the eigenvalues of the Jacobian matrix for the corresponding system linearized about the steady state. These diagrams were obtained using the XPPAUT package by Ermentrout [35]

In the absence of noise, the deterministic ML model can be tuned into conditions for both a Type I and a Type II neuron. For the Type I parameters listed in Table 1, the bifurcation diagram is presented in Fig. 1a (top) together with the *f*–$I_{\mathrm{bias}}$ relationship (bottom). The latter shows a continuous change in the oscillation frequency from zero as $I_{\mathrm{bias}}$ increases beyond a SNIC (saddle-node on an invariant circle) bifurcation point. The SNIC point is located at $I_{\mathrm{SNIC}}\approx 37.7{\text{ μA/cm}}^{2}$. For the Type II parameter values, the bifurcation diagram and the *f*–$I_{\mathrm{bias}}$ relationship are shown in Fig. 1b. A saddle-node (SN) bifurcation on the periodic branch occurs at $I_{\mathrm{SN}}\approx 67.31{\text{ μA/cm}}^{2}$. A subcritical Hopf bifurcation (HB) occurs at $I_{\mathrm{HB}}=68.05{\text{ μA/cm}}^{2}$. The transition from a steady state to an oscillatory state can occur at both the SN and the HB points. The oscillatory solutions that emerge in each case have a finite, nonzero frequency as can be seen in the lower panel of Fig. 1b. 

**Table 1:** Parameters of Type I and Type II models

Shared parameter		Different parameters					
	Values		Type I	Type II		Type I	Type II
$v_{K}$	−84 mV	$g_{K}$	8 mS/cm^2^	5 mS/cm^2^	$I_{\mathrm{bias}}$	33 μA/cm^2^	63 μA/cm^2^
$v_{L}$	−60 mV	$g_{L}$	2 mS/cm^2^	3 mS/cm^2^	$I_{\mathrm{SNIC}}$	37.7 μA/cm^2^	
$v_{\mathrm{Ca}}$	120 mV	$g_{\mathrm{Ca}}$	4.4 mS/cm^2^	5.6 mS/cm^2^	$I_{\mathrm{SN}}$		67.31 μA/cm^2^
*c*	20 μF/cm^2^	$v_{3}$	12 mV	−4.5 mV	$I_{\mathrm{HB}}$	77.62 μA/cm^2^	68.05 μA/cm^2^
$v_{1}$	−1.2 mV	$v_{4}$	17.4 mV	15 mV	*τ*	7 ms	3 ms
$v_{2}$	18 mV	*ϕ*	1/15 ms^−1^	0.04 ms^−1^	$I_{c}$	4.3 μA/cm^2^	4.1 μA/cm^2^

**Fig. 2:**
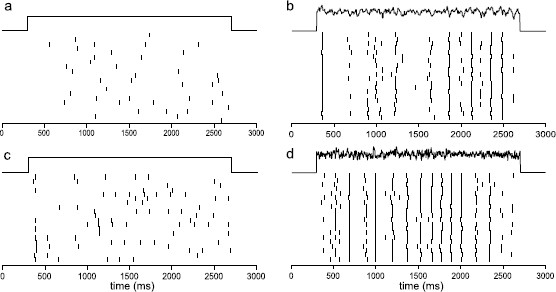
Raster plots showing spike-time reliability (STR) of the ML model in the Type I conditions (*upper panels*, **a**–**b**) and the Type II conditions (*lower panels*, **c**–**d**). Parameter values used are given in Table 1. The coefficient $\delta_{1}$ of the intrinsic noise is 5 μA/cm^2^ for (**a**)–(**d**), and the coefficient $\delta_{2}$ of the external noise is 0.91 μA/cm^2^ in (**b**) and (**d**)

**Fig. 3:**
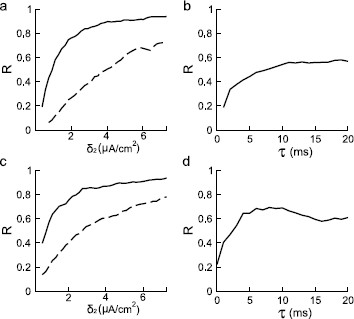
Reliability *R* of the Type I (**a**) and Type II (**c**) ML models is plotted in *the left columns* against the SD of the external noise that is either convoluted (*solid*) or white (*dashed*). *R* is plotted against *τ* for Type I (**b**) and Type II (**d**) in *the right columns*. Parameter values are given in Table 1. The coefficient $\delta_{1}$ of the intrinsic noise is 2 μA/cm^2^ for **a**–**b**, and 5 μA/cm^2^ for **c**–**d**. The coefficient $\delta_{2}$ of the external noise is 0.91 μA/cm^2^ in **b** and 1.64 μA/cm^2^ in **d**

**Fig. 4:**
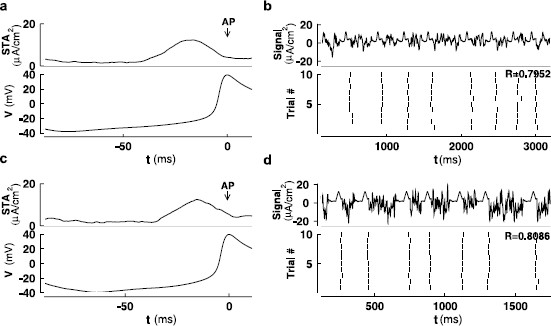
Spike triggered averages (STAs) for Type I (**a**) and Type II (**c**) ML models together with the corresponding response in membrane voltage. Artificial signals are generated (*the upper panel* in **b** and **d**) by connecting many copies of the STA with pieces of background fluctuations of different lengths that are known to be incapable of generating a spike. A typical response of a Type I (**b**) or a Type II (**d**) ML model to such a signal is shown in a raster plot. Noise coefficients $\delta_{1}$ and $\delta_{2}$ for Type I and Type II are as in Figs. 3b, d. The reliability measure *R* is calculated and marked in the figure for each type

**Fig. 5:**
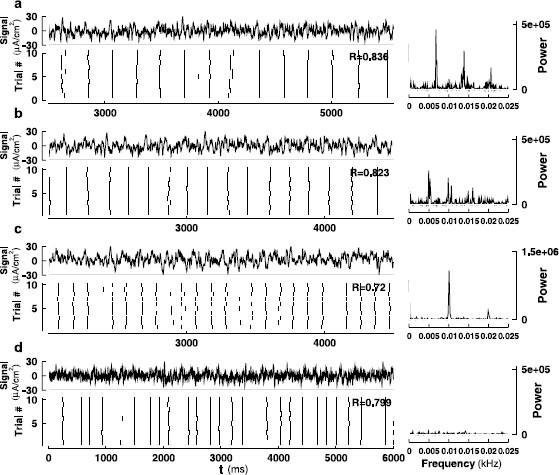
Reliability is insensitive to the frequency content of the noise signal when ISIs are long, shown for Type II. Test noise signals are generated by connecting distinct samples of spike-evoking epochs (SEEs) with intervals of samples that are known to be incapable of generating a spike. The power spectrum for each signal is plotted in *the right panel*. From top to bottom, the peak frequency component is located at 0.00641 kHz in **a**, 0.004 kHz in **b**, 0.01 kHz in **c**, and is insignificant in **d**. The values of *R* are calculated with data collected from 100 trials, each containing more than 45 spikes

**Fig. 6:**
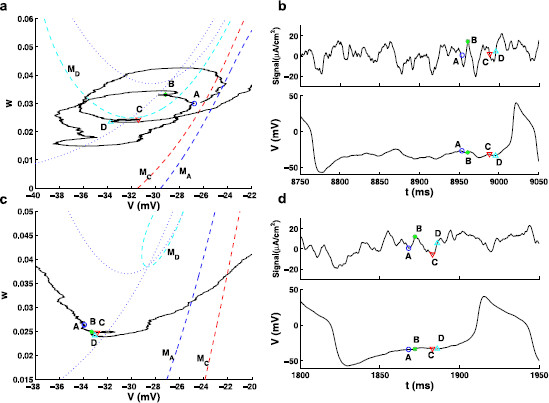
Phase plane trajectories traced out by the responses of the model to two different SEEs (panels **a** and **c**) and the SEEs profiles (*top*) and the corresponding voltage responses (*bottom*) (panels **b** and **d**). Four time points *A*, *B*, *C*, and *D* are chosen and the corresponding location of the (pseudo-)slow manifolds (*dashed lines*) $M_{A}$, $M_{C}$, and $M_{D}$ are plotted in **a** and **c**. *Dotted lines* are nullclines when $I=I_{\mathrm{bias}}+I_{\mathrm{ext}}$. Note that the location of the points *A*–*D* vary around the location of the slow manifold in Type I (*top row*), while for Type II (*bottom row*) these values fluctuate less, thus yielding values of the response well below the (pseudo-)slow manifold as the input signal strength increases for Type II. Note in particular that the value of *w* for Type II, point *D*, is well below the values on the pseudo-slow manifold $M_{D}$. Noise coefficients $\delta_{1}$ and $\delta_{2}$ for Type I and Type II are as in Figs. 3b, d

**Fig. 7:**
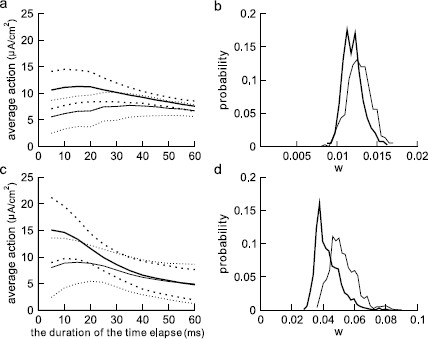
Average action in progressively shorter time intervals before the spike threshold is reached for both Type I (**a**) and Type II (**c**) ML models. *The horizontal axis* represents the duration of time over which action is calculated, starting at the time when spiking threshold is reached. The shorter the time interval, the closer it is to the threshold. *The thick solid curve* represents the average action of reliable SEEs and *the thick dotted curves* represent the upper and lower limits based on the SD. *The thin solid curve* denotes the average action of unreliable SEEs and *the thin dotted curves* mark the upper and lower limits of the SD. *The thick solid curves* are averages of 400 reliable SEEs and *the thin solid curves* are averages of 600 unreliable SEEs. The histogram of the values of the gating variable *w* when the reliable (*bold line*) and unreliable (*thin line*) spike trajectories pass through the threshold at $v_{\mathrm{th}}=-20\text{ mV}$ are plotted in **b** and **d** for Type I and II models, respectively. Noise coefficients $\delta_{1}$ and $\delta_{2}$ for Type I and Type II are as in Figs. 3b, d

**Fig. 8:**
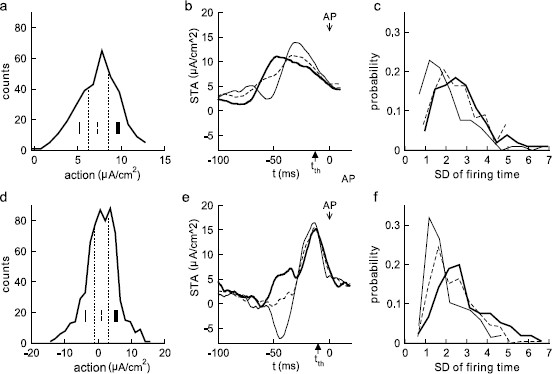
The distributions of action values over a brief time interval of 20 ms for all reliable SEEs. Plotted in **a** and **d** are the action distributions for Type I (*top row*) and Type II (*bottom row*) models, respectively. The distribution is equally divided into three subclasses. The STA of each subclass is shown in **b** and **e** with different line types each marking one subclass as in **a** and **d**. The histograms for the standard deviation of the spike times over all 40 trials for each subclass of reliable SEEs, as identified in panels **a** and **d**, are shown in **c** and **f**. Note that the subclass of reliable SEEs with lower actions typically have smaller standard deviations (SDs) of firing times, thus indicating more closely synchronized spike time reliability. Noise coefficients $\delta_{1}$ and $\delta_{2}$ for Type I and Type II are as in Figs. 3b, d

The Type I and Type II behaviors with additive noise were studied in [31], where computations of the stationary densities and potential stochastic bifurcations were analyzed. While there are no noise-induced bifurcations in both types, additive noise drives spiking in the excitable regime, where $I_{\mathrm{bias}}$ is below the critical bifurcation value and the attracting state is a stable equilibrium. In Type I, the spiking occurs through a coherence-resonance like phenomenon where the excursions follow heteroclinic orbits between an equilibrium and a saddle point. The behavior is similar for Type II, but the excursions follow unstable limit cycles. The simulations in the present study are carried out under the following conditions. Near the Type I transition, we picked $I_{\mathrm{bias}}=33{\text{ μA/cm}}^{2}$ and an external current injection with $I_{c}=E[I_{\mathrm{ext}}]=4.3{\text{ μA/cm}}^{2}$. The total external current, $I_{\mathrm{tot}}=I_{\mathrm{bias}}+I_{\mathrm{ext}}$, has an average amplitude that is equal to $E[I_{\mathrm{tot}}]=37.3{\text{ μA/cm}}^{2}$, which is still below $I_{\mathrm{SNIC}}$. As shown in Figs. 2a and b, a constant input ($\delta_{2}=0$) with this amplitude generates unreliable spikes while a fluctuating input ($\delta_{2}\neq 0$) with identical expected value can generate a train of spikes with rather reliable timing. Near the Type II transition, we picked $I_{\mathrm{bias}}=63{\text{ μA/cm}}^{2}$ and $I_{c}=E[I_{\mathrm{ext}}]=4.1{\text{ μA/cm}}^{2}$ such that $E[I_{\mathrm{tot}}]=67.1{\text{ μA/cm}}^{2}$ which is also below $I_{\mathrm{SN}}$ and $I_{\mathrm{HB}}$. In this case, a constant input ($\delta_{2}=0$) can generate a train of spikes but with unreliable timing (Figs. 2a, c). When replaced by a fluctuating input ($\delta_{2}\neq 0$) with identical expected value, the timing of the spikes triggered by the signal becomes more reliable (Figs. 2b, d). In Fig. 2, we use $\delta_{1}=5$ and $\delta_{2}=0.91$ for both Types for illustration purposes. In the following figures, we use $\delta_{1}=2$ and $\delta_{2}=0.91$ for Type I, and $\delta_{1}=5$ for $\delta_{2}=1.64$ for Type II, as indicated in the captions of Figs. 3, 4, and 6, 7, 8. For Fig. 5, the signal is composed of SEEs and other epochs that do not trigger a spike, constructed in a way to provide certain spectral properties. 

### 2.2 Computational Methods

#### 2.2.1 A Measure for Spike Time Reliability

In the present study, a correlation based measure [32] is used to determine spike time reliability, which is defined as

(2.2) $$R=\frac{2}{N(N-1)}\sum_{i=1}^{N}\sum_{j=i+1}^{N}\frac{\vec{s_{i}}\vec{s_{j}}}{\left|\vec{s_{i}}||\vec{s_{j}}\right|}$$

where *N* is the number of trials and $\vec{s_{i}}$ (i=1;…;N) are the filtered spike trains, that is the convolution of the spike train of a trial and a Gaussian filter with a filter width of $\sigma_{c}=20\text{ ms}$. *R* ranges from 0 (nonreliability) to 1 (full reliability). This correlation based reliability from trial to trial has been used by many other studies (e.g., [2,8,18]), and we would expect similar results for other correlation based measures. For example, (2.2) has a strong correlation (0.96) with the reliability measure based on time series variance presented in [4]. Other STR measures based on the largest Lyapunov exponent (see, e.g., [9]), used in networks, and the phase measure based on the period of the input [7] used in the self-sustained oscillatory regime, do not suit the context of the fluctuation-driven firing regime studied here. 

This reliability measure changes as the number *N* changes. However, in simulations carried out in this study, *R* usually settles to a constant level for values of *N* larger than 30 (results not shown), with remaining parameters unchanged. Therefore, for each *R* value, we calculated in the results section, we chose N=45 to ensure that changes in *R* are not due to changes in *N* in this range.

#### 2.2.2 Simulation of Stochastic Differential Equations

The stochastic model equations in the present work are numerically solved using MATLAB. To reach a good balance between accuracy and computational efficiency, the fourth-order Runge–Kutta scheme is often used for neuron models. The evolution of the deterministic terms of the equations are calculated using a fourth-order Runge–Kutta scheme with a fixed step of Δt=1/30 ms, while the influence of the noise terms is renewed at each time step Δ*t* based on the nature of the noise. Noting that (2.1) has the form

(2.3) $$\begin{array}{rl} c\frac{dv}{dt} & =F(v,w)+I_{\mathrm{bias}}+I_{c}+\delta_{1}\eta_{1}(t)+\delta_{2}\eta_{2}(t),\\ \frac{dw}{dt} & =G(v,w), \end{array}$$

then the increment in the *V* equation takes the form

(2.4) $$V_{n+1}=V_{n}+\frac{\Delta t}{6}(k_{1}+2k_{2}+2k_{3}+k_{4})+\delta_{1}\Delta \eta_{1}+\delta_{2}\Delta \eta_{2},$$

where $k_{j}$ are the standard contributions from a Runge–Kutta 4 (RK4) [33] method for $V^{\prime }(t)=F(v,w)+I_{\mathrm{bias}}+I_{c}$. The white noise increment $\Delta \eta_{1}$ term is distributed as a Gaussian random variable with mean 0, variance $\sqrt{\Delta t}$ and the increment of the external stochastic signal $\eta_{2}$ is the convolution of a white noise *ξ* and an alpha function,

$$\eta_{2}(t)=\int_{0}^{\infty }\xi (t-T)\alpha (T)dT$$

Then $\Delta \eta_{1}$ is simulated by $\sqrt{\Delta t}Z$, for *Z* a standard normal random variable and

(2.5) $$\Delta \eta_{2}=\int_{0}^{\infty }\alpha (T)\left[\Delta \xi (t-T)\right]dT$$

where Δ*ξ* is a white noise increment, Gaussian with mean zero, variance Δ*t*. The increment in the *w* equation is composed simply of the contributions from the RK4 method, as there are no stochastic contributions there. As noted above, $\eta_{2}$ is a frozen copy across trials of the stochastic part of the extrinsic input, while the intrinsic noise $\eta_{1}$ varies from trial to trial.

#### 2.2.3 Computation of Probability Values (p-Values)

p-Values are used to statistically quantify the significant difference of the means of two groups of data in order to determine if the data share the same source [34]. In order to examine characteristics that can be used to differentiate between reliable and unreliable SEEs, we calculate the p-values to measure the average level of difference of “action” levels, as shown in Sect. 3.4, using the paired t-test (due to their time-related properties). The p-value measures whether the data from reliable and unreliable SEE is significantly different, with a small *p* value, normally p<0.05, indicating a small probability that the data from the two classes of SEE’s have the same means. Using the t-test, we also calculated p-values for the values of *w* observed at firing as generated from reliable spikes and unreliable spikes, and the standard deviation of the firing times for different subgroups of reliable SEEs, as shown in Sects. 3.4 and 3.5.

#### 2.2.4 Slow Manifolds and Pseudo-slow Manifolds

As discussed further in Sect. 3.4, it is valuable to compute an invariant manifold with XPPAUT [35], in order to identify the slow or pseudo-slow manifolds that can play a role in understanding the transition from quiescence to spiking. As can be seen from Fig. 1a, under the Type I conditions, the model has three fixed points with the middle fixed point an unstable saddle. Normally, a slow manifold refers to the unstable invariant sets of this saddle point in the phase plane. The Type II setting has only one stable fixed point (see Fig. 1b); however, there exists a well-defined separatrix when the Type II model is in the excitable regime associated with a subcritical Hopf bifurcation as shown in [25] where it is referred to as a threshold manifold. This separatrix has the same function as the slow manifold in the Type I model, thus here we call it a pseudo-slow manifold. The pseudo-slow manifold separates the phase space into two regions, and the dynamics evolve in opposite directions in the two regions on either side of it. In one region, the trajectories flow to the stable fixed point (i.e., rest potential) quickly without firing; in the other region, the trajectories follow a large excursion (i.e., action potential) before returning back to the stable fixed point. Since XPPAUT plots the trajectories of the Type II model automatically, we chose the trajectory that separates the phase space into two regions as described above as the pseudo-slow manifold. The stochastic stimulus drives the voltage dynamics, so that the voltage nullcline (dV/dt=0) varies with time as does the corresponding (pseudo-)slow manifold. Figure 6a and c provide snapshots of the (pseudo-)slow manifold when the injected current has different values at different times, as discussed further in Sect. 3.4. 

## 3 Results

### 3.1 Reliability as a Function of Signal Strength and Correlation Time

Reliability of both the Type I and Type II ML models are studied for increasing input signal strength as measured by the SD of the external input (see Figs. 3a and c). The solid curves represent the case when the noisy signal is generated by convolving a Gaussian white noise and an alpha function with a time constant *τ*. Therefore, *τ* can be regarded as a measure of the correlation time of the input signal. In both Type I and Type II neurons, *R* increases monotonically as the input amplitude increases, consistent with previous studies. Close to full saturation is obtained for SD >20 μA/cm^2^ for both types. Figure 3 also shows the reliability measure as a function of the correlation time when the convoluted input is considered for both the Type I (b) and the Type II (d) models. Reliability for the Type II model shows a local maximum near a *τ* value of about 8 ms (Fig. 3d). This is consistent with previous results obtained in both experiments and simulations [8], with typically a less strong peak for *R* as a function of *τ* for lower intrinsic frequencies. There is no maximum as a function of *τ* for the Type I model, where *R* shows a monotonic increase as *τ* increases (Fig. 3b).

When white noise with zero correlation was used instead, good reliability could also be obtained (dashed curves in Figs. 3a and c). But a larger SD value is required if one wants to achieve the same level of reliability. This suggests that higher noise intensity helps improve the reliability. However, at identical noise intensity correlated noise leads to higher reliability. An optimal correlation time exists for the Type II model. The mechanism underlying the improved reliability at higher values of the correlation time is not known. However, one potential explanation is provided later in this paper.

### 3.2 Spike Triggered Averages Are Effective in Triggering Reliable Spikes

The spike triggered averages (STA) are obtained by averaging many time-varying stimuli in a small time window preceding every spiking event [24]. The averaging process over a large population of stochastic stimulation epochs cancels out the temporally changing components that a spike does not prefer, leaving the optimal signal for a neuron response. Thus, STAs have been widely used to study the sensory filter properties of neurons in auditory neurons [36,37], electrosensory systems [38,39] and even in visual systems [40,41]. 

Here, the spike triggered averages (STAs) are calculated for both the Type I and Type II models over a time duration of 100 ms. Specifically,

(3.1) $$\mathrm{STA}(t)=\frac{1}{N}\sum_{i=1}^{N}\left[u(t-t_{i}+\Delta_{\mathrm{STA}}t)-u(t-t_{i})\right]I_{\mathrm{ext}}(t)$$

where *N* denotes the number of spikes, $t_{i}$ is the spike time, $\Delta_{\mathrm{STA}}t$ is the binwidth of STA, u(t) is the Heaviside unit step function (0 if t<0 and 1 if t≥0). The STA for each type is calculated using 195 SEEs taken from some test signals. Many copies of the STA for each type are then connected by background fluctuations of different lengths that are not capable of triggering a spike. Figure 4 shows that the STAs inserted in these background signals are effective in triggering spikes reliably. Special care was taken to guarantee that the average value of these signals is not altered by such connections between pieces of signals.

### 3.3 The Frequency Content Is not Essential for STR Provided ISIs Are Long

A number of previous works, both experimental and computational, have demonstrated the significance of the frequency content in the external signal to achieve reliable timing in spikes [3-5,18,42]. Here, we demonstrate that under the conditions of STR in an otherwise quiescent neuron, the frequency content is not of special significance for reliability. This is because the stochastic signals we generated in the present study only trigger spike trains with ISIs that are long as compared to the intrinsic IRP. When ISIs are too short, spike time reliability is typically reduced [6] since an SEE is more likely to interact with larger fluctuations at the beginning of the IRP, resulting in a reduced ability of the SEE to trigger a spike. We examine the impact of different frequencies in the input signal in our context, motivated by previous observations that the existence of a significant component of the intrinsic frequency in the signal typically enhances the STR through a resonance effect. In the Type I model, no intrinsic frequency is defined in the vicinity of the SNIC since periodic solutions start with a frequency that is equal to zero. Therefore, we focus on the Type II model. Two intrinsic frequencies can be defined in the vicinity of the Hopf point. The intrinsic frequency of the linearized system for $I_{\mathrm{tot}}=67.1{\text{ μA/cm}}^{2}$ is 0.00715 kHz. The frequency for the stable periodic solution at $I_{\mathrm{tot}}=67.1{\text{ μA/cm}}^{2}$ is close to 0.00641 kHz. These two frequencies become identical at the HB point in this particular case (see Fig. 1b).

Input signals with very different spectral components are tested in the Type II setting, of which four are shown in Fig. 5. These signals were generated as follows. We stimulated the model neuron with 5 segments of convoluted external noise (with τ=3 ms), each 10000 ms long. We picked a total of 112 SEEs and 45 epochs that typically cannot trigger a spike. By connecting these epochs in different combinations, we were able to generate test signals with different spectral content, each one 6000 ms in duration. Figure 5a shows the response to a test signal with a spectral peak at the intrinsic frequency. This is achieved by connecting different SEEs at almost regular intervals that is close to the intrinsic period. This signal triggered a train of spikes with highly reliable spike timing. Figure 5b shows a case where a reliability that is almost identical to the previous case is obtained by a signal with a much less concentrated spectrum. In this case, the highest peak of the spectrum occurs near f=0.0044 kHz. Figure 5c contains another case in which the spectrum is highly concentrated at one single frequency that is equal to 0.01 kHz, which is far from the intrinsic frequency. Reliability remains high in this case although slightly reduced due partly to the shortening of ISIs when the frequency is higher than the intrinsic frequency. In the case shown in Fig. 5d, there is no obvious peak in the spectrum when it is plotted using the same vertical scale as in a and b. However, the reliability remains close to 0.8 in this case.

These results suggest that to achieve high reliability in the noise-induced spike train, there is no need for the signal to contain a major fraction of the Fourier components with frequencies that are near or identical to the intrinsic frequency of either the subthreshold state or the oscillatory state. This result typically applies to the situation when the ISIs in the signal-induced spike trains are relatively long, as in the context of quiescent neurons considered here.

### 3.4 Reliable SEEs on Average Show Accelerated Increase in Action

By focusing on stochastic signals that trigger spike trains with relatively long ISIs, we can ask the following important questions. What are the features that separate the epochs of the signal that trigger a spike with reliable timing from those that cannot? Let us call the SEEs of the signals that reliably drive spiking “reliable SEEs” and those that do not reliably drive spiking the “unreliable SEEs.” We aim to answer the following question. Is there a unique, dominant feature that separates the reliable and unreliable SEEs? The answer to the latter question is probably no, if one examines simply the time series or traces of the two groups SEEs. When a large number of SEEs are examined, they all appear very different from one another, so it is not obvious what features may be appearing more frequently in the reliable SEEs (see for example the two SEEs in Figs. 6b, d). However, comparing distributions of key features of the SEEs indicates a direction to partly answer this question.

For the purpose mentioned above, we need a simple measure to segregate the reliable SEEs from those that are unreliable. We seek an event-based definition. An SEE is called reliable if the spikes it triggers over 30 trials are distributed within a time interval that is smaller than 20 ms. An SEE is regarded as unreliable if the spikes it triggered over 30 trials are spread over a time interval that is larger than 20 ms, in a range between 23 to 115 ms. The cutoff of 20ms was chosen as a natural measure for clearly characterizing the SEE’s, as follows from observations. This measure allows us to set up a database for both reliable and unreliable SEE pools. A total of 450 reliable SEEs and 600 unreliable ones were collected. This leads us to the study of the following features of each SEE.

We also need to identify a critical threshold for providing a clear identification of the initiation of a spike. For a deterministic Type I neuron, the threshold can be clearly defined in terms of the slow or invariant manifold associated with a saddle point. In contrast, in a Type II neuron this threshold has to be determined by finding a separation of the phase space, dividing those trajectories evolving towards a stable fixed point and those following a larger excursion corresponding to firing. Without sufficient input current, the trajectory can not transition from the quiescent region to the firing region, so the (pseudo-)slow manifold here serves as the firing threshold. Both the slow and pseudo-slow manifolds are found with XPPAUT, as described in Sect. 2.2. Since the input current is fluctuating, the (pseudo-)slow manifold is also fluctuating, calculated at a specific time, with the given value of the input at that time. This movement is highlighted in Fig. 6 where dashed lines indicate the slow (Type I) and pseudo-slow (Type II) manifolds, that shift with fluctuations in the input. It is also useful to identify a working threshold in *v* only, that can be used to compare the behaviors for the two types of neurons. With the presence of noise shifting the (pseudo-)slow manifolds, there is some complication in setting a common value of $v_{\mathrm{th}}$. For simplicity, we chose $v_{\mathrm{th}}=-20\text{ mV}$ as this working threshold, where the slope of v(t) turns significantly positive preceding each spike (see Figs. 4a, c), for both Type I and Type II neurons.

The fluctuations of the (pseudo-)slow manifolds as shown in Fig. 6 indicate how the fluctuations in the input signal can promote the transition to firing. These curves also illustrate important differences between Type I and Type II dynamics. For example, by comparing the points A, B, C, and D in the input signal, in the voltage response, and in the phase plane, in Type II neurons we see less fluctuation in the values of *v* and *w* as the input signal fluctuates and drives also fluctuation in the pseudo-slow manifold. For Type I neurons, there is more variation in the response values, which tend to take values closer to the slow manifold, even as it is fluctuating.

One of the main differences between a reliable and an unreliable SEE is the increase in the average “action” level over progressively shorter time intervals immediately preceding the time the spiking threshold is reached. This action level is calculated as the average amount of current delivered during that time interval, i.e., the area under the SEE divided by the length of the interval. On average, the action level of reliable SEEs is significantly higher as the threshold is approached as shown in Figs. 7a, c (p<0.01 for both types). Notice that the average action level of reliable SEEs (thick solid curve) during a brief time interval (say 20 ms long) before the spiking threshold is significantly higher than the corresponding average action level of unreliable SEEs (thin solid curve) for both the Type I and Type II models. As the time interval is pushed further back into the past, the difference between the two is reduced more and more until it disappears at about 40 ms and beyond. This means that when a history longer than 40 ms is taken into account, the difference between the average action levels of reliable and unreliable SEEs is minimal. This reduced difference for longer periods before the spike is a property one would expect for a system exhibiting STR. It suggests that the memory for past events does not last more than 40 ms. 

The increase in action levels of the reliable SEEs (thick curve) continues almost all the way to about 5 ms before reaching the threshold for the Type II model. For this type, the action for the unreliable SEEs (thin curve) also increases as the threshold is approached, reaching a maximum at about 15 ms and starts to decrease for shorter time intervals. For the Type I model, the increase in the thick curve is less steep and reaches a plateau around 15 ms before the spiking threshold. For this type, the thin curve for unreliable SEEs started decreasing at about 35 ms before the spike threshold is reached.

By comparing a large number of SEEs, we found that the responses of a Type II model to reliable SEEs in the phase plane are regularly characterized by a lower value of the gating variable *w* at the moment the voltage variable crosses the working threshold at $v_{\mathrm{th}}=-20\text{ mV}$. This lower value of *w* is then below the pseudo-slow manifold, and thus in a region where the dynamics move easily to increased *v* with increased chance for escape to spiking. Phase plane trajectories traced out by the responses of the model to two different SEEs are plotted in Figs. 6a, c. The corresponding SEEs and the voltage changes in time are shown in Figs. 6b, d, with top figures Type I, bottom figures Type II. Figure 6 also illustrates that pseudo-slow manifolds shift with the input signal, which shows some similarity to the experimental observations [43,44] in which the voltage “threshold” changes with the random gating of the ${\mathrm{Na}}^{+}$ channel. The time series shows the increase in *v* leading to spiking that follows point D, where *w* takes a lower value and the trajectory moves into a range where there is no strong attraction to the fixed point of the underlying deterministic system. The histograms shown in Figs. 7b and d suggest that, on average, the value of the gating variable *w* as the voltage passes through the threshold of $v_{\mathrm{th}}=-20\text{ mV}$ is significantly lower for reliable SEEs (thick curve) than that of the unreliable ones (thin curve) for both Type I and II (p<0.01 for both types).

This observation suggests that the unreliable spikes are triggered at larger values of *w* on average after spending more time in the close vicinity of the pseudo unstable manifold. Also, there is a larger relative shift between the densities of *w* of the reliable and unreliable spikes of Type II, where the difference in means for the two groups is larger than one standard deviation. This result, together with the observations that the response values tend to fluctuate near the slow manifold in Type I and that spiking occurs for slightly lower action values as shown in Fig. 7, suggests that the threshold crossing related to firing in the Type I neuron may be more dependent on the signal amplitude. We discuss this further in Sect. 3.5, in the context of the standard deviation of firing times.

### 3.5 The Influence of Time Profiles Revealed by Individual Features of Reliable SEEs

By studying specific examples of reliable SEEs defined above, one realizes immediately that they still appear very different from each other. This motivated us to divide the reliable SEEs further into three subclasses each one third in numbers: the high action, the medium action, and the low action ones (see the histograms Figs. 8a, d). The goal is to find out if the time profile of an SEE plays a role in determining the reliability of spike timing and, if the answer is positive, what time profile is more favorable for each type. 

The precision of the timing of spikes triggered by individual SEEs is shown in the histograms in Fig. 8c (Type I) and Fig. 8f (Type II), and we relate that precision to the time profile. In these panels, the distributions of the three subclasses are given for the standard deviation (SD) in the spike times triggered by the SEEs over 40 different trials. A larger value of SD corresponds to a lower precision in spike timing. Notice that the highest precision is achieved by the one-third of SEEs with the lowest action levels, for both Types I and II. By plotting the temporal profiles of the spike-triggered averages (STAs) of the three subclasses (see Figs. 8b, e), one notices that the temporal profile of the low action STA is characterized by a stereotypical wave form of a downward change followed by a steep upward swing. This is true for both Type I and Type II models. The profile for the STA of the low action SEEs is consistent with the stereotypical STAs observed in a number of experimental studies [1,6,8]. This suggests that, with a more favorable wave form, an SEE is capable of triggering spikes with higher spike time precision even though its action is in the lowest one-third among all the reliable SEEs. This result also suggests that the spike-triggered average of all SEEs, including both reliable and unreliable ones (see Figs. 4a, c), does not likely possess the most favorable time profile of an SEE that triggers the spikes with most reliable timing.

Comparison of the precision of the low action SEEs (thin line) and high action SEEs (thick line) show significant differences in the standard deviation of the firing times for both types (p<0.01). Observations about the distribution of the action, time profile, and firing times shown in Fig. 8 and time profile in b, e can also be connected with the observations from the phase plane. Time profiles with a downward change can encourage the dynamics to shift toward the steady state, thus settling the response and removing the memory of earlier stimuli. The steep increase of the signal following this downward change then shifts the (pseudo-)slow manifold up, allowing the possibility of a rapid transition to firing. SEEs with high action typically push the response over the firing threshold, but in a way that the dynamics fluctuate around the (pseudo-)slow manifold, resulting in more variation of the location and timing of the transition to firing.

From Figs. 8a, d, we see that the reliable SEEs for Type II neurons have a distribution of action levels with a significantly larger variance (with a probability below 0.01 for the F statistic comparing variances of Figs. 8a, d). Comparing the distribution of the SDs for the firing times shown in Figs. 8c, f, we see that while there is not a significant difference between types for the means of these distributions (p>0.1), the distribution for Type II is somewhat shifted to lower SDs. For example, there is typically 5–10 % more of observed SDs of firing times below the cutoffs in the range 1.5<SD<2 for the subclasses of Type II, as compared with the corresponding Type I subclasses.

The wave form of the thin curves in Figs. 8b and e occurs more frequently when an appropriate correlation time *τ* is used in the convolution. This brings our discussion back to the problem proposed previously. Which features of the SEEs are crucial for STR? The answer is probably a combination of the action level immediately preceding the time when the spiking threshold is reached and a stereotypical wave form of the SEE. We believe the influence of the correlation time *τ* is probably indirect, making it more likely for the stereotypical wave form to occur at increasing values of *τ*.

## 4 Discussion

STR is a complex dynamic phenomenon that depends on both the features of the input signal and the intrinsic properties of the neuron. In a carefully designed study of spike initiation by a current injection in the form of a Gaussian white noise [1], it was revealed that a wide variety of current wave forms could be effective in triggering a spike reliably. It was therefore concluded that a number of stimulus parameters, including polarity, amplitude, variability, slope, acceleration, and temporal correlation, are relevant in spike triggering. It was believed that the absence of one feature in one particular spike-evoking epoch (SEE) of an input signal may be compensated for by the presence of another. Temporal profiles of a SEE favorable for precise spike-generation should be related to the dimensionality of the equations required to describe the dynamics of a neuron and the geometric structure of the manifolds in the phase space that define the thresholds beyond which a spike is generated. The nature and the magnitude of intrinsic noise also play important roles in the reliability of spike timing. These intrinsic properties of a neuron in an experimental setting are typically unknown. This is where computational models, combined with analysis of dynamical structure and comparison of statistical quantities, are helpful for revealing these properties and potentially the roles of different channels in shaping such a favorable profile. 

There is a large number of neuronal types where STR has been observed, ranging from neocortical neurons [2] to neurons in visual cortex [3], motor neurons [1,4,45], mitral cells [46], pyramidal cells, and interneurons [42,47]. This variety has motivated different model choices for computational and theoretical studies of STR, ranging from integrate-and-fire [48], conductance-based models [18], theta-neurons [9], and combinations of these [10]. Although a large number of theoretical works have been devoted to the study of STR, a general theory that explains all observed features of STR remains elusive. Here, we were partly motivated by the work of Tateno and Robinson [15], showing that the regular spiking (RS) pyramidal neurons exhibit a Type I continuous *f*–$I_{\mathrm{bias}}$ relationship while the fast-spiking (FS) interneurons show a Type II discontinuous *f*–$I_{\mathrm{bias}}$ relation. As a result, the RS neurons show properties of a rate-code integrator while the FS neurons behave like resonators controlling the coherence of synchronous firing. We aimed at studying a few important features of STR using a mathematical model that has both Type I and II properties, with the goal of revealing some similarities and differences between neurons that differ in threshold dynamics. Although results presented in this work were obtained in a rather simple, two-variable current based model of neurons and with specific additive internal and external noise inputs, the main conclusions are strikingly similar to the experimental data obtained in *Aplysia california* abdominal ganglia [1]. The action explanation proposed here is very similar to the experiments in which Gaussian white noise with very different amplitudes (38 and 17 nA, respectively) were applied to the neurons, noting that the action level of the corresponding STAs only differ by 14 %. This led to the following conclusion: “when evaluating the spike triggering effectiveness of different waves forms, one must decide on criteria by which to describe and compare them: results …suggest that the amount of delivered charge is a defensible choice.” In that study, “the amount of delivered charge” is identical to our definition of action. 

Comparisons between the Types I and II indicate some key features that contribute to STR. On one hand, one can relate STR to the fact that the system contains some kind of a threshold. A fluctuating stimulus that is frozen across trials yields threshold crossings that are more robust with occasional large amplitude fluctuations, thus making the spike timing more reliable. This is particularly true when the intrinsic noise is relatively small. This observation is consistent both with the monotonic increase in reliability as a function of noise intensity (Fig. 3) and, with the typically increased reliability of SEEs related to higher action levels. In both types, we observe this increase in action level as the voltage approaches the spiking threshold in a reliably reproducible spike. On the other hand, dynamic properties of a neuron sometimes make it respond in an amplified way to certain stimulus profiles. In a recent study, Paydafar [22] showed that a specific wave form of noise facilitates the switch between a stable fixed point and a stable periodic solution. This helps explain why SEEs with certain time profiles are more favorable for inducing reliably timed spikes. Results in Fig. 8, obtained by subdividing the individual “reliable” SEEs into subgroups with different temporal profiles, suggest that, among all the reliable SEEs, the one-third that has lower action level, but with a more favorable time profile actually triggers spikes with higher precision (Fig. 8). While this relationship between action level and precision is observed for both types, the two types differ in the magnitude of the increased action level before the spike, the distribution of observed action levels, the distribution of the values of the gating variable *w* when the voltage reaches its threshold, and shifts in the precision distributions for the reliable SEEs.

The significance of the shape of the time profiles of SEEs is also clearly revealed. A STA with a characteristic downward bias followed by a swift upward swing was found when a depolarizing d.c. was present. A similar profile was also found in [6,8]. This STA is similar in shape to the favorable profiles shown in Fig. 8 in our study. The importance of higher action immediately preceding a threshold value was also demonstrated both in the STA profiles and in the minimal standard deviation for that element of the SEEs. Unfortunately, the SEEs found in these experiments were not further subdivided into reliable and unreliable ones to further confirm the existence of favorable temporal profiles. The remarkable agreements between these experimental data and our model results seem to suggest that the two mechanisms demonstrated here are probably of more general relevance than the model itself. High action basically confirms that robust threshold crossing is possible and temporal profile sensitivity is a clear indication that a low amplitude fluctuation should still be able to trigger spikes reliably provided that the response is amplified through a time localized resonance process. Differences in the underlying dynamical structure relate to the favorable time profiles. In Type II, the variation in the state variables relative to the pseudo-slow manifold is more prominent, leading to reliable responses over a larger range of action. In Type I, there is not a strong deviation from the pseudo-slow manifold, so that initiation of the spike has a greater dependence on the action and signal amplitude rather than temporal profile. The influence of correlation *τ* of the extrinsic noise was also seen to improve reliability through increasing the likelihood of the favorable temporal profiles. As the conclusions made in this study are statistical in nature by averaging over many spike triggering events, the approach is relevant to both experimental and computational studies and could be of general interest to a wide variety of audiences. While reliable spike-evoking epochs on average have higher action, further dividing them into subgroups revealed the sensitivity of STR to temporal profiles of the signal. This is particularly visible in Type II neurons. The connection between these two conclusions is observed when regarded in a statistical context.

It is also important to contrast two different situations under which STR can occur: (i) in neurons that are spontaneously spiking, and (ii) in neurons that are quiescent in the absence of external input. In the first situation, input signals containing the intrinsic frequency of the neuron can trigger spike trains with more reliable timing [4,5]. It has been emphasized in a number of studies that STR is closely related to the fact that the input signal possesses a spectrum that contains a significant fraction of frequency modes that are identical to an intrinsic frequency, with a range of computational and experimental results that explore mechanisms that contribute to low- or high-pass filtering of the input [48,49]. The theory that predicts the emergence of noise-induced negative Lyapunov exponent in noise-driven synchrony between uncoupled phase oscillators [12] provides a rather convincing theoretical explanation for the underlying mechanism. A phase analysis in a more general setting [50] provides consistent and complementary results for noise-driven synchrony in oscillators in an active state analogous to (i). The computational study of [18] considered sinusoidal stimuli in (i) and (ii), termed mean-driven and fluctuation regimes, respectively, focusing on the significance of frequency in the spiking probability and precision. There it was noted that noise could enhance reliability in the fluctuation-driven setting, and that for moderate amplitudes of the sinusoidal stimuli, the most reliable response rate selected a frequency resonant with the subthreshold voltage oscillations. 

To show that the frequency content of the stimulus is not always of central importance, we aimed at studying STR in an idealized and simplified model in situation (ii), quiescence in the absence of external input. In this setting, without strong, high frequency inputs, the spike trains triggered by the stochastic signals contain long ISIs only. This makes the frequency content of the input signal of little importance for STR (Fig. 5). In response to stochastic external inputs, both the Type I and Type II neurons are capable of generating trains of spikes with reliable spike timing. In both types, the reliability measure *R* shows monotone increase as the intensity of the noisy input increases. When *R* is plotted as a function of the correlation time of the extrinsic input, Type II shows a local maximum while Type I gives only a monotone increase.

For the second situation (ii), however, the phase theory alone does not apply, as indicated by the study of conditional oscillators synchronized through a common noisy input signal [19]. There it was shown that a combined phase and amplitude analysis is needed to completely describe the phenomenon. In addition, [51] explores scenarios where geometry and phase-space structures play a critical role, so that perturbative approaches based on phase response curves can not predict the dynamical behavior. Since the approach of [19] does not apply to full spiking induced by noise, a corresponding theory is still needed for such a case. Mechanisms for spike initiation by subthreshold fluctuations are probably of crucial importance in such a theory. Encouraging progress has been made toward understanding these mechanisms based on the concept of “feature detection” [20]. The ideas for key features for spiking combined with the stochastic theory for the synchrony of two uncoupled, noise-induced coherent oscillators driven by a common noise input in [19] should open up promising directions toward a theoretical explanation of STR in the case of underlying quiescence. 

The results for neurons that are quiescent in the absence of an external input suggest a number of other future directions for investigation. For example, experimental and computational studies have explored STR in networks, exploring the effects of network interactions, coupling, and different sources of heterogeneity. In a study of single and two-layered networks of theta neurons [9], STR was analyzed using Lyapunov exponents and synaptic variance in the context of local noise, trial-to-trial variation affecting only select neuron, and global noise, trial-to-trial variation as an input to the entire network. A recent computational study of network dynamics indicates mechanisms that allow reliability to appear even in systems where chaotic dynamics are prominent, also suggesting that certain classes of initial states may play an important role [52]. In [19], specific results for synchronized and phase-locked responses were obtained for different relative strengths of global and local noise, including some first steps in spatial heterogeneity of the noise. However, the concepts of action and time profiles have not been analyzed in the network case, and heterogeneity of coupling and neural properties have not been analyzed in the case where the underlying state of the network is quiescence. Experimental results in [53] showed that network interactions enhance the frequency range of reliable responses, in the context where the networks are in an active state without the (noisy) external input. The question remains whether similar network tuning of otherwise quiescent neurons increases the reliability, or if the same insensitivity to signal frequency is observed for the network as in the single quiescent neuron case considered in the present work. 

## Competing Interests

The authors declare that they have no competing interests.

## Authors’ Contributions

NY did all computational simulations. YXL and RK both initiated the research project, and all authors participated in all discussions during the progress of the project. YXL initiated the writing of the manuscript, both RK and NY were active participants through out the writing process.
